# Predicting Severity of Acute Pancreatitis—Evaluation of Neutrophil-to-Lymphocyte Count Ratio as Emerging Biomarker: A Retrospective Analytical Study

**DOI:** 10.7759/cureus.74881

**Published:** 2024-11-30

**Authors:** Akhil Vincent, Shashirekha C A

**Affiliations:** 1 General Surgery, Sri Devaraj Urs Medical College, Kolar, IND

**Keywords:** acute pancreatitis, ct severity index, inflammatory biomarkers, neutrophil-to-lymphocyte ratio (nlr), severity prediction

## Abstract

Introduction

Acute pancreatitis (AP) is a pancreatic inflammatory disease that can range in severity from mild, self-limiting forms to severe cases with high mortality rates. AP has various etiologies, including lifestyle factors like alcohol consumption and obesity, and its rapid progression makes early and accurate prediction of severity critical for effective management and improved patient outcomes.

The traditional AP severity assessment tools, such as Ranson's criteria and APACHE II, require extensive data and time, making them less feasible in emergency settings. In response, simpler biomarkers that can quickly predict AP severity upon patient presentation are needed to enable early risk stratification and targeted interventions.

The study aims to address this research gap by evaluating the neutrophil-to-lymphocyte ratio (NLR) as a potential biomarker for predicting AP severity, as well as assessing its correlation with the CT Severity Index, a widely used measure of AP severity.

Methods

The study used a retrospective analytical design, conducted at the R L Jalappa Hospital & Research Centre in Karnataka, India. The researchers included 118 patients diagnosed with acute pancreatitis (AP) according to the Revised Atlanta Classification.

The dataset collected from the participants' medical records included variables such as age, gender, history of alcohol and tobacco use, duration of abdominal pain, ICU stay, CT Severity Index scores, and the neutrophil-to-lymphocyte ratio (NLR).

Statistical analysis was performed using SPSS software version 21.0 (IBM Corp., Armonk, NY, USA). A p-value of less than 0.05 was considered statistically significant.

This comprehensive methodological approach aimed to provide precise insights into the role of NLR in predicting AP severity while accounting for variability in patient data.

Results

The study included 118 patients, with 85 classified as having mild to moderate pancreatitis and 33 with severe pancreatitis. There were no significant differences between the two groups in terms of demographic factors such as gender, BMI, alcohol use, smoking, and comorbidities.

The study also examined the relationship between the neutrophil-to-lymphocyte ratio (NLR) and the CT Severity Index, a measure of pancreatitis severity. The results showed a strong positive correlation between NLR and the CT Severity Index (r = 0.860, p < 0.001). This indicates that higher NLR values are associated with more severe pancreatitis, as measured by the CT Severity Index.

These relationships suggest that NLR reflects the inflammatory response in acute pancreatitis, with higher levels of inflammatory markers associated with elevated NLR values.

Conclusion

This study aimed to evaluate the neutrophil-to-lymphocyte ratio (NLR) as a biomarker for predicting the severity of acute pancreatitis (AP). We conducted a retrospective analysis of 118 AP patients, categorizing them into mild-to-moderate and severe groups. NLR was significantly higher in the severe AP group compared to the mild-to-moderate group, suggesting its potential as an early predictor of AP severity. The study also examined the correlation between NLR and the CT Severity Index, a widely used measure of AP severity, further supporting the utility of NLR as a rapid and accessible tool for risk stratification in AP management.

## Introduction

Abdominal discomfort that appears suddenly and ranges in intensity from minor to life-threatening is a symptom of acute pancreatitis (AP) which is a pancreas inflammatory disease. When digestive enzymes start working in the pancreas instead of the small intestine, inflammation results, in damaging tissue and perhaps harming adjacent organs [1]. AP has a global rate of approximately 13 to 45 cases per 100,000 people annually, with variations observed across geographic regions and population groups [2]. The severity of AP can range from mild, self-limiting forms to severe cases, which may cause systemic complications, organ failure, or even fatality. The mortality rate is about 5% for mild to moderate cases, while it increases drastically to 20-30% in severe forms with complications such as necrotizing pancreatitis [3].

The AP pathogenesis is complex, involving inflammatory mediators, cytokines, and activated enzymes, all of which contribute to tissue injury and the systemic inflammatory response [4]. AP can arise from various etiologies, including gallstones, chronic alcohol use, hypertriglyceridemia, and infections. Recently, there has been an increment in cases associated with lifestyle factors such as alcohol consumption and obesity, due to the rising load of the disease [5]. Considering the condition's propensity for fast development and the significant morbidity and mortality associated with severe variants, early and precise prediction of AP severity is critical. Early diagnosis of patients at risk of serious AP might lead to more effective care techniques, thereby lowering mortality and improving patient outcomes Predictive Tools and Challenges.

A multitude of grading methods and biomarkers have been developed over time to help identify high-risk individuals and assess the severity of AP. Traditional grading methods such as Ranson's criteria, the Acute Physiology and Chronic Health Evaluation II (APACHE II) score, and the Bedside Index for Severity in Acute Pancreatitis need extensive laboratory data and time, limiting their value in emergency situations. Another popular method for estimating the severity of an illness that uses laboratory and physiological data is the APACHE II score [6]. Though APACHE II has been validated in various conditions beyond AP, it remains complex and labor-intensive, as it includes 12 variables assessed over time, making it cumbersome for routine clinical practice [7]. Furthermore, both Ranson's and APACHE II scores require a minimum of 48 hours to yield reliable predictive insights, which could delay early intervention in patients with rapidly progressing severe AP.

In response to these limitations, the BISAP score was developed as a simpler, more accessible tool for predicting AP severity within the first 24 hours of admission [8]. BISAP uses five parameters - blood urea nitrogen, struggling mental state, systemic inflammatory response syndrome, age, and pleural effusion - to estimate the risk of complications. This system offers the benefit of being less time-consuming and has shown predictive accuracy similar to APACHE II. However, BISAP still requires specific laboratory values and clinical observations, making it less feasible in resource-limited settings where rapid access to comprehensive testing may not be available.

Given the limitations of traditional scoring systems, there is a clear need for simpler, cost-effective, and rapid biomarkers that could aid in the prompt prediction of AP severity, particularly in emergency and resource-constrained environments. Many studies are currently exploring alternative markers that can predict severity without the complexity or time burden associated with existing systems. A biomarker that can quickly predict AP severity upon patient presentation would significantly improve clinical outcomes by enabling earlier and more targeted interventions [7].

Emerging biomarkers - neutrophil-to-lymphocyte ratio (NLR)

The neutrophil-to-lymphocyte ratio (NLR) is a promising biomarker to predict the extent of AP since it indicates the balance between lymphocyte-mediated immunological regulation and neutrophil-mediated inflammation. The NLR [9] is the proportion of the absolute neutrophil count to the absolute lymphocyte count, which is frequently measured in complete blood counts. Increased neutrophil counts are typically indicative of acute inflammation, while reduced lymphocyte counts can signal a compromised immune response, both of which are relevant to the inflammatory profile of AP [10].

The utilization of NLR as a prognostic marker in AP continues to gain traction because of its ease of use, access, and quick availability via standard blood testing. Unlike traditional scoring systems, NLR does not require a range of laboratory parameters or physiological data, making it a potentially useful tool in both well-equipped and resource-limited healthcare settings [9]. Recent studies have shown that a higher NLR is associated with more severe forms of AP and increased mortality, suggesting its role in early risk stratification for AP patients [10, 11]. Additionally, NLR has been evaluated in other inflammatory conditions such as cardiovascular disease and cancer, where it has shown potential as a predictor of disease outcomes, further supporting its relevance in AP severity prediction [12].

The simplicity of calculating NLR, combined with its ability to reflect both inflammation and immune responses, positions it as a promising alternative or adjunct to traditional severity scoring systems. This marker’s feasibility for early-stage risk stratification in AP presents significant advantages, potentially enabling clinicians to initiate aggressive management earlier in high-risk patients. However, while NLR shows promise, further research is necessary to establish standardized cutoff values for NLR in AP severity prediction, as current studies indicate variability based on population and clinical context [11].

Research gap and study rationale

Despite the growing body of literature supporting NLR’s predictive value in AP, gaps remain in the standardization and generalization of its use as a biomarker. Most traditional scoring systems are cumbersome and require comprehensive clinical and laboratory data that may not be readily available in all settings. NLR, on the other hand, is a straightforward and readily accessible marker with the potential to overcome these limitations. Nevertheless, its adoption in clinical attempts is still constrained by the lack of standardized thresholds for differentiating between mild, moderate, and severe AP cases. Current research stresses the necessity for prospective findings to validate the predictive accuracy of NLR across different populations and clinical settings [13]. Moreover, comparative studies examining the predictive efficacy of NLR alongside established scoring systems like APACHE II and BISAP would provide valuable insights into its utility in routine clinical practice. Understanding the variations in NLR across diverse AP patient profiles and integrating NLR with other emerging biomarkers may also enhance its predictive power, paving the way for more nuanced and efficient AP severity assessment tools [14].

This study, therefore, aims to address this research gap by evaluating NLR as a capacity biomarker for forecasting AP severity in a retrospective cohort of patients. By examining the relationship between NLR and traditional severity indices, such as the CT severity index, this research seeks to establish a more accessible, timely, and cost-effective predictive tool for AP. Identifying an effective biomarker that can be readily utilized upon patient admission holds promise in improving early risk stratification, thereby facilitating quicker clinical decision-making and potentially enhancing patient outcomes in AP management.

Objectives

The major goal of this research is to assess the predictive value of the Neutrophil-to-Lymphocyte Ratio (NLR) in predicting the degree of severity of acute pancreatitis (AP). The secondary objective is to assess the correlation between NLR and the CT Severity Index, which could support early decision-making in AP management.

## Materials and methods

Study design

The research was carried out at the R L Jalappa Hospital & Research Centre in Karnataka and used a retrospective analytical design. An extensive examination of the neutrophil-to-lymphocyte ratio (NLR) as a likely predictor of the seriousness of acute pancreatitis (AP) was made possible by the inclusion of 118 individuals with AP diagnoses.

Inclusion & exclusion criteria

To guarantee the precision and dependability of the results, patients were chosen according to precise inclusion and exclusion criteria. Patients aged 18 and above who had been identified with acute pancreatitis in accordance with the Revised Atlanta Classification satisfied the inclusion criteria. Patients excluded from the research included those over 80 years of age, those with a diagnosis of cancer, or those undergoing treatment for hematological proliferative diseases. These criteria were designed to reduce confounding factors that might interfere with the relationship between NLR and AP severity.

Data collection

Clinical and demographic data were collected from each participant’s medical records. The dataset included variables for example age, gender, history of alcohol and tobacco use, duration of abdominal pain, ICU stay, CT Severity Index scores, and the neutrophil-to-lymphocyte ratio. This comprehensive data collection aimed to capture a wide range of factors that could influence AP outcomes and the severity classification.

Statistical analysis

Statistical analysis was conducted to compare categorical and continuous variables between mild to moderate and severe AP groups. Categorical variables were inspected by the chi-square test; however, when the expected frequency was below 5, Yates' correction was applied to ensure accurate results. Quantitative variables were first assessed for normality using the Kolmogorov-Smirnov test. Only certain variables - haematocrit, white blood cell (WBC) count, neutrophil, and lymphocyte counts - followed a normal distribution. To compare mild to moderate and severe pancreatitis in these normally distributed variables, an independent t-test was used. For the rest of the non-normally distributed quantitative variables, the Mann-Whitney U test was used. Furthermore, Spearman Rank correlation was used to assess the association between the CT Severity Index and NLR with other factors. The statistical analysis was performed using SPSS software version 21.0 (IBM Corp., Armonk, NY, USA), with a p-value of less than 0.05 indicating statistical significance. This comprehensive approach to methodology was designed to give precise insights into NLR's involvement in forecasting AP severity while also accounting for variability in patient data.

## Results

In this study, 118 patients were analyzed, of whom 85 were categorized with mild to moderate pancreatitis and 33 with severe pancreatitis. Table 1 summarizes the general demographic and clinical characteristics, and there were no statistically significant variations between groups in terms of gender, body mass index (BMI), history of alcohol use, smoking, or comorbid conditions such as diabetes mellitus (DM), congestive cardiac failure (CCF), obstructive airway disease, and coronary artery disease (CAD) (p > 0.05 for all variables). These findings suggest that these baseline demographic factors do not substantially differentiate severity groups, indicating that factors other than general demographic and lifestyle variables may be more predictive of pancreatitis severity.

**Table 1:** General profile of the study population ns: non-significant; All of them have abdominal pain. BMI: Body Mass Index; DM: Diabetes Mellitus; CAD: Coronary Artery Disease

Characteristics	Category	Mild to moderate pancreatitis (n=85)		Severe pancreatitis (n=33)		Total	P-value
		No	Per cent	No	Per cent		
Gender	Female	12	85.7	2	14.3	14	0.344^ns^
	Male	73	70.2	31	29.8	104	
BMI	Normal	32	78.0	9	22.0	41	0.526^ns^
	Overweight	24	66.7	12	33.3	36	
	Obese	29	70.7	12	29.3	41	
Alcohol use	No	13	81.3	3	18.8	16	0.559^ns^
	Yes	72	70.6	30	29.4	102	
Smoking	No	12	80.0	3	20.0	15	0.669^ns^
	Yes	73	70.9	30	29.1	103	
DM	No	67	72.0	26	28.0	93	0.997^ns^
	Yes	18	72.0	7	28.0	25	
Congestive Cardiac Failure	No	75	69.4	33	30.6	108	0.060^ns^
	Yes	10	100.0	0	0.0	10	
Obstructive Airway Disease	No	76	73.1	28	26.9	104	0.711^ns^
	Yes	9	64.3	5	35.7	14	
CAD	No	81	73.0	30	27.0	111	0.398^ns^
	Yes	4	57.1	3	42.9	7	

Table 2 presents a comparison of quantitative variables between the mild to moderate and severe pancreatitis groups, highlighting significant differences in several physiological and biochemical parameters. The mean age was notably lower in the severe pancreatitis group (43.21 ± 23.12 years) compared to the mild to moderate group (54.61 ± 16.67 years), with a statistically significant p-value of 0.012, suggesting a potential association between younger age and increased severity of pancreatitis. Respiratory rate also showed a significant difference, with the mild to moderate group presenting a higher average rate (26.77 ± 4.64 breaths/min) compared to the severe group (24.70 ± 4.91 breaths/min, p < 0.05).

In terms of vital signs and hematological parameters, several variables showed significant distinctions. Heart rate was considerably elevated in the severe group (107.24 ± 15.45 beats/min) compared to the mild to moderate group (98.37 ± 15.64 beats/min, p < 0.01). The severe pancreatitis group had significantly higher white blood cell (WBC), neutrophil, and monocyte counts (p < 0.01 for each parameter), while the lymphocyte count was significantly lower (0.91 ± 0.24) than the mild to moderate group (1.24 ± 0.39, p < 0.01). The severe pancreatitis group had a substantially higher neutrophil-to-lymphocyte ratio (NLR) (13.04 ± 2.72) relative to the mild to moderate group (7.28 ± 1.89), with a p-value < 0.01, demonstrating a robust link between higher NLR and severity of pancreatitis. In short, the analysis of quantitative variables indicates that younger age, higher heart rate, increased WBC, neutrophil, and monocyte counts, as well as a higher NLR, are associated with more severe pancreatitis. These findings underscore the potential utility of these variables, particularly NLR, as indicators of disease severity, supporting further investigation into their predictive value for clinical outcomes in acute pancreatitis.

**Table 2:** Results of comparison of quantitative variables between mild to moderate pancreatitis and severe pancreatitis ** Significant at 0.01 level; * Significant at 0.05 level; ns: non-significant WBC: White Blood Cells; CT Severity Index: Computed Tomography Severity Index; NLR: Neutrophil-to-Lymphocyte Count Ratio

Variables	Total (n=118)	Mild to moderate pancreatitis (n=85)	Severe pancreatitis (n=33)	P-value
Age (Years)	51.42 ± 19.29	54.61 ± 16.67	43.21 ± 23.12	0.012*
Days In Pain Before Admission	2.64 ± 1.33	2.54 ± 1.14	2.91 ± 1.72	0.530^ns^
Respiratory Rate Breaths (Min)	26.19 ± 4.79	26.77 ± 4.64	24.70 ± 4.91	0.029*
Systolic BP (mmHg)	121.78 ± 13.56	122.12 ± 14.07	120.91 ± 12.34	0.617^ns^
Diastolic BP (mmHg)	79.75 ± 7.89	80 ± 8.17	79.09 ± 7.23	0.529^ns^
Heart Rate Beats (Min)	100.85 ± 16.03	98.37 ± 15.64	107.24 ± 15.45	0.010**
Temperature (Degree Celsius)	36.98 ± 0.68	36.96 ± 0.68	37.02 ± 0.70	0.458^ns^
Amylase (UL)	685.23 ± 391.46	700.97 ± 451.97	644.7 ± 147.32	0.446^ns^
Lipase (UL)	368.56 ± 85.92	375.46 ± 89.82	350.79 ± 73.21	0.744^ns^
Haematocrit	39.40 ± 10.98	39.35 ± 10.84	39.55 ± 11.49	0.817^ns^
Serum Creatinine (mgdL)	1.03 ± 0.32	1.05 ± 0.32	0.99 ± 0.31	0.417^ns^
WBC (10^3^ µL)	11.75 ± 2.66	11.15 ± 2.63	13.29 ± 2.08	<0.001**
Neutrophil (10^3^ µL)	9.42 ± 2.57	8.67 ± 2.39	11.34 ± 1.97	<0.001**
Lymphocyte (10^3^ µL)	1.15 ± 0.39	1.24 ± 0.39	0.91 ± 0.24	<0.001**
Monocyte (10^3^ µL)	0.17 ± 0.07	0.16 ± 0.07	0.2 ± 0.08	0.027*
CT Severity Index	4.90 ± 1.94	3.85 ± 1.05	7.61 ± 0.56	<0.001**
NLR	8.89 ± 3.36	7.28 ± 1.89	13.04 ± 2.72	<0.001**

**Figure 1:**
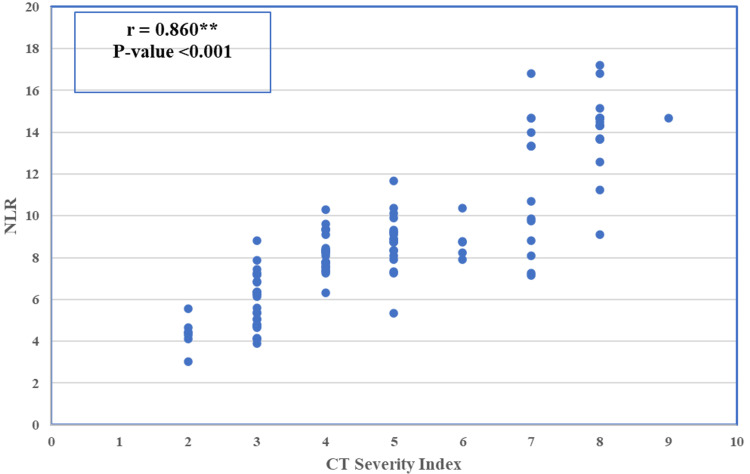
Positive association between the CT Severity Index and the Neutrophil-to-Lymphocyte Ratio (NLR) (Spearman correlation coefficient = 0.860, p-value < 0.001)

Figure 1 shows a substantial positive association between the CT Severity Index and the Neutrophil-to-Lymphocyte Ratio (NLR) (Spearman correlation coefficient = 0.860, p-value < 0.001). This significant positive association indicates that when NLR rises, so does the CT Severity Index, meaning that individuals with a higher NLR have more severe acute pancreatitis as evaluated by CT imaging. This correlation underscores the potential of NLR as a biomarker for assessing disease severity in clinical settings.

Table 3 provides additional correlation data between the CT Severity Index, NLR, and various clinical variables. Age and lymphocyte count had a significant negative connection with the CT Severity Index (r = -0.205, p < 0.05 and r = -0.487, p < 0.01, respectively), indicating that older age and larger lymphocyte counts are related to lower severity scores. White blood cell (WBC) count, neutrophil count, and monocyte count had a positive correlation with the CT Severity Index (r = 0.516, r = 0.623, and r = 0.224, accordingly, all with p < 0.05), indicating that higher levels of these variables correspond to higher severity.

**Table 3:** Correlation of other clinical variables with CT severity index and NLR ** Significant at 0.01 level; * Significant at 0.05 level; ns: non-significant WBC: White Blood Cells; NLR: Neutrophil-to-Lymphocyte Count Ratio

Variables	CT Severity Index	NLR
Age	-0.205*	-0.147
Days In Pain Before Admission	0.140	0.128
Respiratory Rate Breaths (Min)	-0.148	-0.064
Systolic BP (mmHg)	-0.141	-0.125
Diastolic BP (mmHg)	-0.112	-0.045
Heart Rate Beats (Min)	0.111	0.131
Temperature (Degree Celsius)	0.046	0.056
Amylase (UL)	-0.018	0.029
Lipase (UL)	-0.030	-0.01
Haematocrit	0.081	0.058
Serum Creatinine (mgdL)	-0.151	-.194*
WBC (10^3^ µL)	0.516**	0.519**
Neutrophil (10^3^ µL)	0.623**	0.608**
Lymphocyte (10^3^ µL)	-0.487**	-0.646**
Monocyte (10^3^ µL)	0.224*	0.242**
NLR	0.860**	1

## Discussion

The demographic and baseline characteristics in this study, such as gender, BMI, alcohol use, smoking, and comorbid conditions (DM, CCF, CAD, obstructive airway disease), showed no significant variances between mild-to-moderate and severe pancreatitis groups. These findings align with prior research by Pang et al. (2018), which also suggested that baseline demographic factors and lifestyle choices are limited in their ability to predict acute pancreatitis (AP) severity [15]. This underscores the need for clinicians to focus on specific physiological and hematological markers when stratifying risk and predicting disease progression in AP patients [16]. The association between younger age and severe AP observed in our study introduces a compelling clinical consideration. Younger patients with AP presented with more severe cases, as indicated by a statistically important difference in age between the severity groups (mean age 43.21 ± 23.12 years in the severe group vs. 54.61 ± 16.67 years in the mild-to-moderate group). While prior studies have typically associated older age with increased mortality in AP [17], our results suggest that younger patients may have unique risk factors or immune response mechanisms that exacerbate disease severity. This observation contrasts with findings from the APPRENTICE registry, which noted a higher average age among severe cases in Europe, possibly due to differing etiologies and comorbid profiles [16]. The younger age profile in severe AP may reflect regional variations in AP etiology, potentially indicating lifestyle or genetic factors contributing to inflammation and immune response in younger populations [18].

Our results highlight the critical role of vital signs and hematological parameters in assessing AP severity. Elevated heart rate was a distinguishing feature of severe AP cases, with patients in this group showing significantly higher heart rates than those in the mild-to-moderate group (107.24 ± 15.45 beats/min vs. 98.37 ± 15.64 beats/min). This supports previous findings where elevated heart rate was linked to systemic inflammation and adverse outcomes in AP [19]. The systemic inflammatory response triggered by AP likely explains this physiological change, as tachycardia serves as an adaptive response to counteract hypotension caused by fluid shifts and immune activation, reinforcing its role as a prognostic marker [20].

Hematological markers further differentiated between severity groups in this study, with severe AP patients exhibiting significantly elevated white blood cell (WBC), neutrophil, and monocyte counts, alongside a lower lymphocyte count. Elevated WBC and neutrophil levels are known indicators of inflammatory activity, as seen in studies by Park et al. (2019) and Zhang et al. (2021), which consistently link heightened inflammatory responses to worse clinical outcomes in AP [18, 19]. The significantly elevated neutrophil-to-lymphocyte ratio (NLR) in severe cases (13.04 ± 2.72 vs. 7.28 ± 1.89 in mild-to-moderate cases) underlines NLR's potential as a robust marker for AP severity, aligning with findings that associate NLR with increased mortality risk in AP and other inflammatory diseases [18].

Additionally, the observed lymphopenia in severe AP cases underscores the immune suppression characteristic of severe inflammation, which has been widely reported as a marker of poor prognosis in AP. Suppressed lymphocyte counts, coupled with high neutrophil levels, suggest an impaired adaptive immune response, further validating NLR as a useful predictor of severe outcomes [17, 20]. These findings are consistent with studies on systemic inflammatory diseases where an elevated NLR is often indicative of an imbalanced immune response, favoring neutrophil-driven inflammation over lymphocyte-mediated regulation [19].

The significant correlation between the Neutrophil-to-Lymphocyte Ratio (NLR) and the CT Severity Index (CTSI) in assessing acute pancreatitis (AP) severity highlights NLR’s role as a non-invasive biomarker. In this study, the Spearman correlation (r = 0.860, p < 0.001) suggests that an elevated NLR is indicative of the immune response shift favoring neutrophil-driven inflammation over lymphocyte-mediated regulation, a balance crucial to severe AP progression. This is consistent with findings by Jeon and Park (2017), where elevated NLRs were linked with higher AP severity and adverse outcomes, particularly organ failure [21]. They identified an optimal cut-off for NLR at 4.76, which reliably distinguished patients at risk of severe outcomes. Other studies, such as those by Kong et al. (2020), reinforce the predictive value of NLR, supporting its use as an efficient early indicator for AP severity due to its correlation with systemic inflammation markers like C-reactive protein (CRP) and its diagnostic performance similar to conventional scoring systems [22].

NLR’s utility extends beyond AP, with its application across inflammatory conditions, as supported by Vemparala et al. (2021), who also emphasized its predictive role in critical outcomes [23]. They found that NLR levels over 10.8 were associated with severe AP, corroborating findings from this study and Jeon and Park’s work [21]. Similarly, Bhanou et al. (2018) illustrated that high NLR levels align with extended hospital stays and intensive care needs, underscoring its relevance in early triage decisions [11]. While NLR is advantageous for its simplicity and rapid accessibility, its accuracy may vary compared to traditional scoring systems such as APACHE II and Ranson’s criteria, which involve more extensive data collection but offer marginally higher sensitivity and specificity in some settings. Nevertheless, NLR’s ease of use supports its integration as a supplementary assessment tool, especially in acute settings where rapid decision-making is paramount.

The study also demonstrated NLR’s significant positive correlations with inflammatory markers such as WBC and neutrophil counts, alongside a negative correlation with lymphocyte counts. This pattern aligns with the findings by Kokulu et al. (2018), who highlighted that NLR reflects both inflammatory and immune system responses, making it a valuable biomarker for AP [10]. While the negative association between NLR and lymphocyte count signals immune suppression, as observed in systemic inflammatory response syndrome (SIRS), the increased neutrophil activity correlates with heightened inflammatory processes. These results align with insights by Junare et al. (2021), who posited that as inflammation worsens, the lymphocyte counts tend to drop, further driving the NLR value up [24]. Such insights validate NLR as a comprehensive indicator of both immune and inflammatory dynamics, supporting its broader use alongside other markers in assessing AP severity [25].

Clinical implications and future applications

Given NLR’s strong association with AP severity and ease of calculation, it emerges as a valuable tool for risk assessment in emergency settings, particularly where resources are constrained. Kong et al. (2020) argued that NLR, due to its high sensitivity and specificity, could be integrated into initial evaluation protocols for AP, enabling healthcare providers to triage patients based on risk levels without the need for immediate CT imaging or complex biochemical markers [22]. This study reinforces these points by demonstrating NLR's high predictive accuracy for AP severity, advocating for its inclusion in initial clinical assessments, which could optimize patient outcomes in both low-resource and high-acuity settings.

In addition to its role in emergency triage, NLR offers the potential for personalized treatment strategies. By identifying high-risk patients early, NLR can facilitate targeted interventions, such as intensive monitoring and early therapeutic adjustments. This concept aligns with the principles of personalized medicine, as proposed by Bhanou et al. (2018), who suggested that NLR can guide tailored treatment regimens in AP management [11]. In cases where elevated NLR signals severe inflammation, clinicians can adapt care plans to focus on mitigating systemic inflammatory effects, reducing the likelihood of complications. From a public health perspective, NLR’s cost-effectiveness and accessibility make it a viable tool for optimizing resource allocation in AP management, especially in settings with high patient loads. Jeon and Park (2017) emphasized the value of NLR in predicting AP severity and guiding early intervention, thus potentially reducing the strain on intensive care units by enabling earlier, less intensive management of patients with lower NLRs [21]. This approach could enhance resource allocation by prioritizing critical care resources for patients most likely to benefit, ultimately improving health outcomes while maintaining cost efficiency.

Limitations

This paper is subjected to many limitations that should be acknowledged. First, as a retrospective analysis, it is inherently constrained by the availability and accuracy of recorded data, which may introduce selection bias and limit generalizability. The single-center design restricts the broader application of findings, as variations in clinical practices and patient demographics may influence the results. Additionally, although the neutrophil-to-lymphocyte ratio (NLR) demonstrated significant associations with acute pancreatitis (AP) severity, the lack of standardized cutoff values for NLR limits its immediate clinical application. Further, this study did not account for potential confounders such as coexisting inflammatory conditions, which may impact NLR levels, thus possibly affecting the precision of severity predictions.

## Conclusions

To sum up, this study's results highlight NLR's potential as a useful and approachable biomarker for predicting acute pancreatitis's severity early on. The strong correlation between elevated NLR and severe AP suggests its utility as an adjunct to conventional scoring systems, particularly in resource-limited settings where rapid, cost-effective diagnostic tools are essential. NLR's predictive accuracy is supported by the study, but further multicenter, prospective research is needed to standardize NLR thresholds and confirm its effectiveness in a range of patient types. Such advancements could facilitate timely clinical decision-making, optimize resource allocation, and potentially improve patient outcomes in AP management.
